# Association between human leukocyte antigens and graft-versus-host disease occurrence after allogenic hematopoietic stem cell transplantation

**DOI:** 10.1590/S1516-31802012000400003

**Published:** 2012-09-04

**Authors:** Daniela Máira Cardozo, Sofia Rocha Lieber, Silvia Barbosa Dutra Marques, Francisco José Aranha, Afonso Celso Vigorito, Cármino Antonio de Souza, Jeane Eliete Laguila Visentainer

**Affiliations:** I MSc. Doctoral Student in Clinical Medicine, Universidade Estadual de Campinas (Unicamp), Campinas, São Paulo, Brazil.; II MSc, PhD. Biomedic, HLA Laboratory, Hematology and Hemotherapy Center, Universidade Estadual de Campinas (Unicamp), Campinas, São Paulo, Brazil.; III MSc. Supervisor, HLA Laboratory, Hematology and Hemotherapy Center, Universidade Estadual de Campinas (Unicamp), Campinas, São Paulo, Brazil.; IV MD, PhD. Hematologist, Hematology and Hemotherapy Center, Universidade Estadual de Campinas (Unicamp), Campinas, São Paulo, Brazil.; V MD, PhD. Titular Professor, Hematology and Hemotherapy Center, Universidade Estadual de Campinas (Unicamp), Campinas, São Paulo, Brazil.; VI MSc, PhD. Adjunct Professor, Department of Basic Health Science, Universidade Estadual de Maringá (UEM), Maringá, Paraná, Brazil.

**Keywords:** Graft vs host disease, Histocompatibility antigens, Hematopoietic stem cells, Polymorphism, genetic, Transplants, Doença enxerto-hospedeiro, Antígenos de histocompatibilidade, Células-tronco hematopoéticas, Polimorfismo genético, Transplantes

## Abstract

**CONTEXT AND OBJECTIVE:**

Graft-versus-host disease (GVHD) is one of the complications following allogenic stem cell transplantation. This study investigated an association between human leukocyte antigen (HLA) and the occurrence of acute and chronic GVHD in patients who had received stem cell transplantations from HLA-identical siblings.

**DESIGN AND SETTING:**

Retrospective study at Hematology and Hemotherapy Center, Universidade Estadual de Campinas (Unicamp).

**METHODS:**

The participants were 176 patients whose first transplant was between 1997 and 2009. HLA genotyping was performed serologically and using the polymerase chain reaction with specific primer sequence.

**RESULTS:**

Acute GVHD was positively associated with HLA-A10 (P = 0.0007), HLA-A26 (P = 0.002), B55 (P = 0.001), DRB1*15 (P = 0.0211) and DQB1*05 (P = 0.038), while HLA-B16 (P = 0.0333) was more frequent in patients without acute GVHD. Chronic GVHD was positively associated with HLA-A9 (P = 0.01) and A23 (P = 0.0292) and negatively with HLA-A2 (P = 0.0031) and B53 (P = 0.0116). HLA-B35 (P = 0.0373), B49 (P = 0.0155) and B55 (P = 0.0024) were higher in patients with acute GVHD grade 3 or above, than in other patients. In patients with extensive chronic GVHD, HLA-A9 (P = 0.0004), A24 (P = 0.0059) and A26 (P = 0.0411) were higher than in other patients, while HLA-A2 was lower (P = 0.0097).

**CONCLUSION:**

This study suggests that HLA can influence the incidence and severity of acute and chronic GVHD. However, a study with a better design and more patients will be needed to confirm these results.

## RESUMO

**CONTEXTO E OBJETIVO:**

A doença do enxerto contra o hospedeiro (DECH) é uma das complicações pós-transplante alogênico de células progenitoras hematopoéticas. Este estudo investigou uma associação entre o antígeno leucocitário humano (HLA) e a ocorrência de DECH aguda e crônica, em pacientes que receberam transplantes de irmãos HLA-idênticos.

**TIPO DE ESTUDO E LOCAL:**

Estudo retrospectivo no Centro de Hematologia e Hemoterapia da Universidade Estadual de Campinas (Unicamp).

**MÉTODOS:**

Os participantes foram 176 pacientes cujo primeiro transplante foi entre 1997 e 2009. A tipagem HLA foi realizada por sorologia e reação em cadeia da polimerase (PCR) com sequência específica de *primers*.

**RESULTADOS:**

A DECH aguda foi associada positivamente com HLA-A10 (P = 0,0007), HLA-A26 (P = 0,002), B55 (P = 0,001), DRB1*15 (P = 0,0211) e DQB1*05 (P = 0,038), enquanto HLA-B16 (P = 0,0333) foi mais frequente em pacientes sem DECH aguda. A DECH crônica foi associada positivamente com HLA-A9 (P = 0,01) e A23 (P = 0,0292) e, negativamente, com HLA-A2 (P = 0,0031) e B53 (P = 0,0116). HLA-B35 (P = 0,0373), B49 (P = 0,0155) e B55 (P = 0,0024) estavam aumentados em pacientes com DECH aguda grau 3 ou maior, em comparação aos outros pacientes. Em pacientes com DECH crônica extensa, HLA-A9 (P = 0,0004), A24 (P = 0,0059) e A26 (P = 0,0411) estavam aumentados em comparação aos outros pacientes, enquanto HLA-A2 estava diminuído (P = 0,0097).

**CONCLUSÕES:**

Este estudo sugere que o HLA pode influenciar a ocorrência de DECH aguda e crônica e a sua gravidade. No entanto, um estudo com melhor desenho e com mais pacientes será necessário para confirmar esses resultados.

## INTRODUCTION

Graft-versus-host disease (GVHD) remains an important source of morbidity and mortality following allogenic hematopoietic stem cell transplantation (HSCT). Although susceptibility to this complication has been shown to be influenced by the immunogenetic background of the donor/recipient pair,^1^^,^^2^ the potential impact of human leukocyte antigen (HLA)-like genes on HSCT outcomes remains poorly explored.

Under these circumstances, donor T-cell recognition of host HLA can give rise to GVHD, and host recognition of donor HLA may increase the risk of graft failure. Identifying GVHD risk factors may help to reduce its incidence and severity. Previous reports have suggested that particular HLA system factors have an impact on GVHD^3^^,^^4^^,^^5^^,^^6^^,^^7^^,^^8^^,^^9^^,^^10^^,^^11^ and give rise to a reduced relapse rate and improved survival after HSCT.^12^

HLA molecules are the most important immunogenic proteins contributing towards the intensity of the GVHD reaction.^13^ Class I antigens (HLA-A, -B and -C) and Class II antigens (DR, DQ and DP) are the classical molecules in the major histocompatibility complex (MHC), which are encoded by MHC in chromosome 6. HLA antigens from host tissues are recognized by donor T cells that are critical for the development of both acute and chronic GVHD.

To our knowledge, in Brazil, there have not been any studies investigating an association between HLA and GVHD development. Consequently, there is intense interest in defining the best HLA genetic markers for allogenic HSCT outcomes and incorporating these into routine donor selection strategies in the Brazilian population.

## OBJECTIVE

The objective of this study was to investigate an association between HLA and the occurrence of acute and chronic GVHD in patients who had received stem cell transplantation from HLA-identical siblings in a Brazilian population.

## PATIENTS AND METHODS

### Study design and data collection

The clinical records of allogenic HSCT recipients in one hematology and hemotherapy center were retrospectively reviewed and 176 patients who had received a first matched-sibling allogenic HSCT between 1997 and 2009 were found. These patients were followed up to ascertain their clinical outcomes up to the cutoff point of June 2010. The characteristics of the study population are presented in Table 1.

**Table 1.** Characteristics of the study population (n = 176)

Characteristic	
Average patient age in years (range)	34.0 (1-60)
Average donor age in years (range)	32.0 (1-62)
Recipient-donor gender - n (%)	
M/M	57 (32.4%)
M/F	51 (29.0%)
F/M	33 (18.7%)
F/F	35 (19.9%)
Diagnosis - n (%)	
AA	23 (13.1%)
PNH	4 (2.3%)
ALL	25 (14.2%)
AML	42 (23.9%)
CML	73 (41.5%)
CMML	2 (1.1%)
MDS	6 (3.4%)
Source of cells - n (%)	
Bone marrow	104 (59.1%)
Peripheral blood stem cell	72 (40.9%)

M = male; F = female; AA = aplastic anemia; PNH = paroxysmal nocturnal hemoglobinuria; ALL = acute lymphoblastic leukemia; AML = acute myeloid leukemia; CML = chronic myeloid leukemia; CMML = chronic myelomonocytic leukemia; MDS = myelodysplastic syndrome.

Only patients who developed acute GVHD or survived for more than 100 days without GVHD, and were therefore at risk of developing acute GVHD, were included in the analyses for acute GVHD. For chronic GVHD, patients who had developed chronic GVHD or survived for more than 110 days without chronic GVHD were included.

All of the patients and donors were residents of Campinas and the surrounding region in the State of São Paulo and were classified as a mixed population due to their genetic background of European, African and Amerindian origin.

This study was conducted in accordance with the guidelines of the Ethics Committee of the University Hospital of Universidade Estadual de Campinas (Unicamp).

### GVHD assessment

Occurrences of GVHD were classified in accordance with Glucksberg et al.^14^ and Atkinson et al.^15^ In our study, we classified grades 0 to II as mild acute GVHD and grades III to IV as severe acute GVHD. Chronic GVHD was considered to be severe when it was classified as extensive.

### HLA genotyping

HLA matching was performed serologically for HLA-A and -B antigens (One Lambda, Canoga Park, CA, USA), and for HLA-DRB1 and -DQB1 using molecular typing (Dynal sequence-specific primer, Dynal Ltd., Bromborough, Wirral, UK; One Lambda, Canoga Park, CA, USA).

### Statistical analysis

Screening for associations between HLA-A, HLA-B, HLA-DRB1 and DQB1 variants and GVHD incidence was carried out using chi-square statistics with Yates correction or Fisher’s exact test when needed. Odds ratios (OR) with 95% confidence intervals (95% CI) were also calculated. P values < 0.05 were considered statistically significant, but to account for multiple comparisons, the observed P values were corrected by means of Bonferroni’s correction. The P value was multiplied by the number of comparisons made. The resulting corrected Pc < 0.05 was accepted as significant. Cumulative incidences were calculated for acute and chronic GVHD.

## RESULTS

### Patients and donors

The patients were 108 men and 68 women with a median age of 34 years (range: 1-60 years) and the donors were 90 men and 86 women with a median age of 32 years (range: 1-62 years). The conditioning therapy, GVHD prophylaxis, GVHD treatment, transplantation procedures and clinical support have been described elsewhere.^16^^,^^17^

### Occurrence of acute and chronic GVHD

In total, 150 patients showed no evidence of acute GVHD or developed acute GVHD grade I; 11 (42.3%) had grade II; 11 (42.3%) had grade III; and 4 (15.4%) had grade IV. The cumulative occurrence of acute GVHD of grades II-IV was 14.8%. A total of 140 patients were evaluated for chronic GVHD: 22 (15.7%) developed the limited and 58 (41.4%) the extensive clinical form. The cumulative occurrence of chronic GVHD was 57.1%.

### Frequency of HLA variants in GVHD

**Figure 1.**
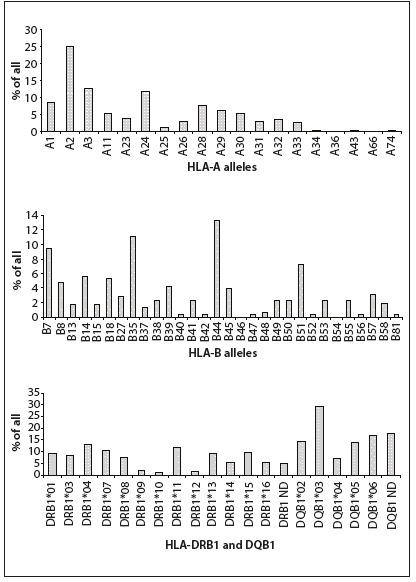
Human leukocyte antigen (HLA)-A, -B, -DRB1 and -DQB1 allele frequencies among 176 hematopoietic stem-cell transplant (HSCT) patients with HLA-identical sibling donors.

The frequencies of the HLA class I and II variants in patients who received a HSCT are shown in Figure 1. By cross-tabulating each HLA variant with the occurrence of GVHD, ten variants were found to be associated with either the presence or the absence of GVHD with P < 0.05. These HLA variants were A2, A9, A10, A23, A26, B16, B53, B55, DRB1*15 and DQB1*05, as shown in Tables 2**and**3. After Bonferroni’s correction, the HLA-A10, -A26 and -B55 variants remained statistically significant.

**Table 2.** Association between human leukocyte antigen (HLA) alleles and acute graft-versus-host disease (aGVHD)

HLA allele	n	aGVHD grades 0-I (%)	n	aGVHD grades II-IV (%)	P	OR	95% CI
	n = 150		n = 26				
A10	7	2.3	7	13.5	0.0007	6.51	2.18-19.44
A26	5	1.7	6	11.5	0.0020	7.70	2.26-26.25
B16	23	7.7	0	0.0	0.0333	0.12	0.01-1.96
B55	2	0.7	5	9.6	0.0010	15.85	2.99-84.07
DRB1*15	25	8.6	9	21.4	0.0211	2.91	1.25-6.77
DQB1*05	38	15.2	12	30.0	0.0380	2.39	1.12-5.11

n = number of alleles; P = probability; OR = odds ratio; CI = confidence interval.

**Table 3.** Association between human leukocyte antigen (HLA) alleles and chronic graft-versus-host disease (cGVHD)

HLA allele	n	cGVHD absence (%)	n	cGVHD presence (%)	P	OR	95% CI
	n = 58		n = 82				
A2	41	35.3	31	18.9	0.0031	0.43	0.25-0.74
A9	10	8.6	34	20.7	0.0100	2.77	1.31-5.87
A23	1	0.9	10	6.1	0.0292	7.47	0.94-59.17
B53	5	4.3	0	0.0	0.0116	0.07	0.0-1.25

n = number of alleles; P = probability; OR = odds ratio; CI = confidence interval.

### Frequency of HLA variants in acute and chronic GVHD

Table 2 shows the significant frequencies of HLA associated with acute GVHD. Acute GVHD (grades 0-I) was positively associated with HLA-A10 (P = 0.0007; Pc = 0.0133), HLA-A26 (P = 0.002; Pc = 0.038), B55 (P = 0.001; Pc = 0.033), DRB1*15 (P = 0.0211; Pc = 0.2743) and DQB1*05 (P = 0.038; Pc = 0.19). HLA-B16 was more frequent in patients who did not develop acute GVHD (grades 0-I), but this difference did not remain significant after Bonferroni’s correction (P = 0.0333; Pc = 1.099).

Chronic GVHD was positively associated with HLA-A9 (P = 0.01; Pc = 0.19) and A23 (P = 0.0292; Pc = 0.5548), and negatively with A2 (P = 0.0031; Pc = 0.0589) and B53 (P = 0.0116; Pc = 0.3828).

### Frequency of HLA variants in severe acute and chronic GVHD

Table 4 compares the frequencies of HLA in relation to severe acute GVHD. It shows that the HLA-B35 (P = 0.0373; Pc = 1.2309), B49 (P = 0.0155; Pc = 0.5115) and B55 (P = 0.0024; Pc = 0.0792) variants were higher in patients with acute GVHD grade 3 or above, than in other patients.

In patients with extensive chronic GVHD, the HLA-A9 (P = 0.0004; Pc = 0.0076), A24 (P = 0.0059; Pc = 0.1121) and A26 (P = 0.0411; Pc = 0.7809) variants were higher than in other patients, as shown in Table 5. HLA-A2 was lower in these patients (P = 0.0097; Pc = 0.1843).

**Table 4.** Results from analysis on severe (grades III-IV) acute graft-versus-host disease (aGVHD)

HLA allele	n	aGVHD grades 0-II (%)	n	aGVHD grades III-IV (%)	P	OR	95% CI
	n = 161		n = 15				
B35	30	9.3	7	23.3	0.0373	2.96	1.17-7.48
B49	4	1.2	3	10.0	0.0155	8.33	1.88-41.52
B55	4	1.2	4	13.3	0.0024	12.23	2.89-51.75

n = number of alleles; P = probability; OR = odds ratio; CI = confidence interval.

**Table 5.** Results from analysis on severe (extensive) chronic graft-versus-host disease (cGVHD)

HLA allele	n	cGVHD absence or limited (%)	n	cGVHD extensive (%)	P	OR	95% CI
	n = 80		n = 60				
A2	51	31.9	21	17.5	0.0097	0.45	0.25-0.81
A9	14	8.8	30	25.0	0.0004	3.48	1. 57-6.91
A24	11	6.9	22	18.3	0.0059	3.04	1.41-6.55
A26	2	1.3	7	5.8	0.0411	4.89	1.00-24.00

n = number of alleles; P = probability; OR = odds ratio; CI = confidence interval.

## DISCUSSION

Many studies have analyzed risk factors for GVHD in relation to HLA, cytokines, killer immunoglobulin-like receptors (KIR) and other variants.^3^^,^^4^^,^^5^^,^^6^^,^^7^^,^^8^^,^^9^^,^^10^^,^^11^^,^^18^^,^^19^^,^^20^^,^^21^^,^^22^^,^^23^^,^^24^

Our analysis investigated the role of class I and II HLA variants on GVHD development in a population of 176 patients, and suggests that these were determinants for acute and chronic GVHD in allogenic HSCT patients with an HLA-identical sibling donor. HLA-A26 (a split of HLA-A10) was associated with higher risk of acute GVHD, in agreement with previous studies.^3^^,^^5^^,^^7^^,^^9^^,^^11^

According to Bross et al.,^8^ HLA-A19 and B17 were associated with a lower risk of acute GVHD, whereas HLA-Cw4, A-11 and B21 appeared to be associated with a higher risk. Our data were concordant with this, showing an association between HLA-B49 (a split of HLA-B21) and severe acute GVHD. However, HLA-B35 was associated with a higher risk of severe acute GVHD, in contrast with the findings of Storb et al. and Ghavamzadeh et al.^5^^,^^10^

We did not find any correlation between acute GVHD risk and HLA-A3 and A11, in contrast with the findings of Clark et al. and Bross et al., respectively;^6^^,^^8^ and likewise in relation to HLA-DR1^6^ and DR3.^7^

While the present study showed that HLA-DRB1*15 and DQB1*05 (one of the alleles in linkage disequilibrium with DRB1*15) were higher in patients with acute GVHD (grades II to IV), Battiwalla et al. identified absence of HLA-DR15 as a factor significantly associated with acute GVHD.^4^ However, there is a hypothesis that immunodominant myeloid antigens are preferentially presented by HLA-DR15, thus suggesting that this allele has an important role in relation to acute GVHD development.

Several factors could explain these differential results, including patients who received transplants from unrelated donors and the use of serological typing methods in a subgroup of patients, whereas all individuals in our cohort received transplants from HLA-identical siblings and were typed as HLA class II using molecular methods. It is worth noting that our study has an important limitation regarding clinical application, due to the statistical method used.

Moreover, Stern et al. reported that DRB1*15 showed an association with lower disease relapse and greater survival in patients treated with HSCT for leukemia or non-Hodgkin lymphoma, but they did not observe any significant difference regarding the incidence of acute GVHD.^12^ In our view, DRB1*15 could reduce the graft-versus-leukemia effect after HSCT, thereby increasing the incidence of GVHD.

Our study also suggested that other HLA antigens were associated with acute GVHD, such as HLA-B16 (protection factor) and B55 (susceptibility factor), which were also associated with GVHD severity.

This study found statistically significant differences in the occurrence and severity of chronic GVHD. HLA-A9 (A23 or A24) was positively associated with both of them, while HLA-A2 was associated with protection. HLA-B53 was associated with chronic GVHD development, but not with the severe (extensive) form, while HLA-A26 was associated with extensive chronic GVHD. Although HLA-A26 has been associated with acute GVHD and severe chronic GVHD, the immunopathogenesis of these GVHD forms are different, which could explain the different associations observed with the forms of disease.

Few studies have investigated an association between HLA and chronic GVHD. Remberger et al. found that HLA-B27 was associated with a lower risk of developing chronic GVHD.^9^ According to Battiwalla et al., there was no significant difference in the two groups, regarding chronic GVHD incidence or severity.^4^

The differences between various HLA variants and their correlations with the risk of GVHD in different centers and countries may be due to differences in ethnicity between populations. In addition to these differences, we emphasize that our study had limitations relating to its design and the number of patients.

## CONCLUSION

These results show that MHC variants may influence the occurrence of GVHD in HSCT with HLA-identical sibling donors. However, we suggest that collaborative studies using larger central registry datasets should be conducted in order to explore clinical outcomes according to HLA antigen status in HLA-matched allogenic stem cell transplantation.
